# Persistence and Progression of Masked Hypertension: A 5-Year Prospective Study

**DOI:** 10.1155/2013/836387

**Published:** 2013-12-17

**Authors:** Xavier Trudel, Alain Milot, Chantal Brisson

**Affiliations:** ^1^Unité de Recherche en Santé des Populations du Centre de Recherche FRQS du CHU de Québec, CHU de Québec, 1050 Chemin Ste-Foy, Québec, QC, Canada G1S 4L8; ^2^Département de Médecine, Université Laval, Québec, QC, Canada G1V 0A6; ^3^Département de Médecine Sociale et Préventive, Université Laval, Québec, QC, Canada G1V 0A6

## Abstract

*Objectives*. To examine masked hypertension persistence over 5 years. *Methods*. White-collar workers were recruited from three public organizations. Blood pressure (BP) was measured using Spacelabs 90207. Manually operated BP was defined as the mean of the first three readings taken at rest. Ambulatory BP was defined as the mean of the next readings taken every 15 minutes and recorded during working hours. BP was assessed three times over 5 years. Masked hypertension was defined as manually operated BP less than 140 and less than 90 mmHg and ambulatory BP at least 135 or at least 85 mmHg. Sustained hypertension was defined as manually operated BP at least 140 or at least 90 mmHg and ambulatory BP at least 135 or at least 85 mmHg or being treated for hypertension. *Results*. BP measurements were obtained from 1669 participants from whom 232 had masked hypertension at baseline. Persistence of masked hypertension was 38% and 18.5%, after 3 and 5 years, respectively. Progression to sustained hypertension was 26% and 37%, after 3 and 5 years, respectively. *Conclusion*. Among baseline masked hypertensives, one-third progressed to sustained hypertension and about one out of five remained masked after 5 years, potentially delaying diagnosis and treatment.

## 1. Introduction

The use of ambulatory blood pressure (BP) monitoring has added a new source of information about out-of-office blood pressure. Discrepancies between office and out-of-office blood pressure have resulted in four potential groups of blood pressure status: normotension, sustained hypertension, white coat hypertension, and masked hypertension. According to international guidelines, elevated daytime ambulatory BP (at least 135 or at least 85 mmHg) in the face of normal office BP (less than 140 and less than 90 mmHg) is defined as masked hypertension (MH) [1, 2]. There has been growing interest in MH over the last decade. Studies have reported that associations between MH and cardiovascular diseases are as strong as those found for sustained hypertension [3]. Yet, MH recognition as a clinical entity of its own is still a matter of debate. One main reason for this is the scarce available evidence supporting its persistence over time.

In one prospective study, conducted among patients retrieved from a hospital-based ambulatory BP monitoring database, 11 out of 25 (44%) initially masked hypertensive patients were still masked when remonitored, while 7 (28%) had sustained hypertension, after a 1.5-year follow-up [4]. A nested case-control study, which has assessed MH among youth [5], has reported that 18 out of 34 baseline masked hypertensives became normotensives, 13 had persistent MH (38%), and 3 (9%) had sustained hypertension, after a 37-month median follow-up. Another study conducted among 50 borderline hypertensives has reported a “fair to moderate” reproducibility of MH, within a 1-week interval (*k* = 0.47) [6]. A fourth study has reported moderate reproducibility of hypertension classification, using morning (*k* = 0.58) and evening (*k* = 0.46) home blood pressure monitoring over 6 months [7]. Finally, two studies conducted among treated hypertensives have reported low MH persistence over one year and 6 months, respectively [8, 9]. It is noteworthy that treated MH qualitatively differs from untreated MH. Elevated ABP in the face of normal clinic BP might be related to poor adherence to treatment or ineffective clinical management explained by the fact that clinic BP is thought to be controlled. Also, to be treated, masked hypertensives must have been identified as having high BP in a clinical setting. Finally, relying on BP levels in treated masked hypertensives to classify them is prone to misclassification, as their blood pressure levels are likely to have been lowered by medication.

Studies about persistence of MH include a wide range of study groups and vary widely in their definitions, equipment used, measurement procedures, and populations. These studies also share some limitations, such as the small number of included participants [4–6] as well as the short follow-up period [4, 6–8]. Therefore, it is difficult to determine the real persistence of MH from the present literature.

The objective of the present study was twofold: (i) to examine, over 5 years, the evolution of MH prevalence in 1669 white-collar workers and (ii) to examine masked hypertension persistence and progression to sustained hypertension, over 5 years, among baseline masked hypertensives.

## 2. Method

### 2.1. Population and Study Design

The study population was composed of white-collar workers from three public insurance institutions. Workers were recruited for a prospective cohort study aiming to measure the effect of psychosocial environmental factors on the evolution of BP over a 5-year follow-up. Participation rate at baseline was 80.4%. Workers were recruited if they were not pregnant, not suffering from cardiovascular disease, and worked more than 20 h per week. Data were collected in three steps: at baseline, after 3 years, and after 5 years. At each time, workers completed a self-administered questionnaire on work characteristics and BP risk factors. Trained staff installed the monitoring device, collected ambulatory BP measurements, and measured weight and height. At baseline, the study sample was composed of 2178 workers. About 23% (*N* = 509) of the eligible workers at baseline were lost during follow-ups. Final study sample thus involved 1669 participants (711 men and 958 women). This study was approved by the ethical review board of the CHU de Québec. Participants provided their written informed consent.

### 2.2. BP Measurements

Blood pressure (BP) was measured using the Spacelabs 90207 oscillometric monitor (Spacelabs Produits Médicaux Ltée, St-Laurent, Quebec, Canada) validated by the independent investigators' protocol, recommended by the Association for the Advancement of Medical Instrumentation and British Hypertension Society [10, 11]. In a research office at the participant's workplace, the device was installed on the nondominant arm if BP difference measured on both arms was inferior to 10 mmHg. Otherwise, it was installed on the arm showing the higher BP level. After the participant had been sitting for 5 minutes, BP was measured three times in the presence of trained personnel using the office check mode of the Spacelabs monitor. Then, BP was measured every 15 min by the same monitor for the rest of the working day. Manually operated BP (MOBP) was defined as the mean of the first three readings taken at rest and displayed by the Spacelabs monitor. Then, ambulatory BP (ABP) was defined as the mean of the next readings taken every 15 minutes and recorded by the same monitor during regular daytime work.

Participants must have been measured at least 20 times, which is a more stringent restriction than the 14 measurements recommended by international guidelines [12]. Participants were classified into four categories according to the European Society of Hypertension guidelines [2]: normotension, in which MOBP was less than 140 and less than 90 mmHg and ABP was less than 135 and less than 85 mmHg; white coat hypertension, in which MOBP was at least 140 or at least 90 mmHg and ABP was less than 135 and less than 85 mmHg; MH, in which MOBP was less than 140 and less than 90 mmHg and ABP was at least 135 or at least 85 mmHg; and sustained hypertension, in which MOBP was at least 140 or at least 90 mmHg and ABP was at least 135 or at least 85 mmHg. Participants treated for hypertension were classified as sustained hypertensives. Workers were systematically informed about their hypertensive status between measurements and referred to their physician when their ABP values were compatible with the diagnosis of hypertension.

### 2.3. Risk Factors

Lifestyle risk factors examined were cigarette smoking status, BMI, alcohol intake, and sedentary behaviours. Smoking status was defined as the daily consumption of at least one cigarette per day. Body weight and height were measured to calculate BMI (kg/m^2^). Alcohol intake was measured using the following three categories, related to weekly intake frequency during the past 12 months: less than one drink per week, one to five drinks per week, and six or more drinks per week. Participants were further classified as having sedentary behaviours (yes/no), according to their weekly leisure physical activity frequency (<1/week/≥1/week). Age, education, and family history of cardiovascular disease (CVD) were also examined. The definition of the latter group was based on the declaration by the participant of a cardiovascular event, such as angina, myocardial infarction, coronary revascularization, or stroke, suffered by their father, mother, brother, or sister before the age of 60. The risk factors listed above were evaluated using validated protocols [13, 14].

### 2.4. Analyses

**Figure 1:**
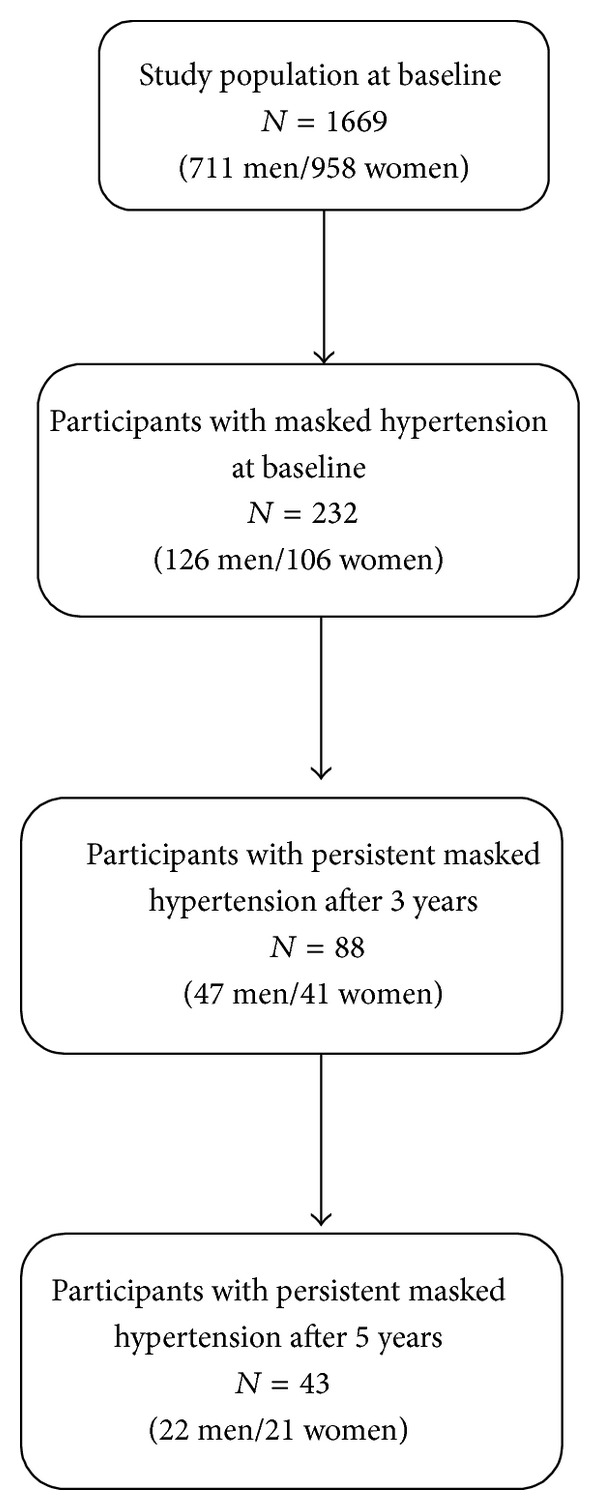
Flowchart of masked hypertension within-subject persistence over time.

All analyses were conducted using SAS [15]. A tabular analysis was first conducted to describe baseline demographic and lifestyle characteristics for all participants. Comparisons between mean BP (MOBP and ABP) were computed using an analysis of variance. Prevalence estimates for each hypertension category were calculated for the whole study population, at each measurement time. Persistence of MH and progression to sustained hypertension were examined in two steps: (1) using the within-subject persistence of MH, after 3 years, among baseline masked hypertensives (*N* = 232) and (2) using the within-subject persistence of MH, from 3 to 5 years, among participants with masked hypertension both at baseline and after 3 years (*N* = 88). The overall persistence of MH after 5 years, among baseline masked hypertensives, was also examined.  Figure 1 presents the selection process used to assess within-subject MH persistence. Progression to sustained hypertension was defined as having MOBP at least 140 and at least 90 mmHg and ABP at least 135 and at least 85 mmHg, or being treated for hypertension.

## 3. Results

Table 1 presents the distribution of baseline demographic and lifestyle characteristics for each hypertension category. MH prevalence was higher among men (17.7%) compared to women (11.1%) and increased with age. MH prevalence was also higher among participants with higher education. Smoking status and sedentary behaviours were not associated with differences among groups (*P* > 0.05), whereas BMI, alcohol intake, and family history of CVD were associated with an increasing proportion of masked hypertension in higher category.

Table 2 presents the mean systolic and diastolic BP, at baseline, by hypertension category and measurement type. In men, those with MH had higher BP levels than normotensive participants for both measurement types, namely, for systolic MOBP, 130.1 versus 123.0 mmHg; for diastolic MOBP, 82.5 versus 75.5 mmHg; for systolic ABP, 134.6 versus 123.3 mmHg; and for diastolic ABP, 87.8 versus 77.9 mmHg. In women, masked hypertensives also had higher BP levels than normotensive, namely, for systolic MOBP, 127.4 versus 116.7 mmHg; for diastolic MOBP, 82.1 versus 73.0 mmHg; for systolic ABP, 132.8 versus 118.1 mmHg; and for diastolic ABP, 87.4 versus 75.7 mmHg.

**Figure 2:**
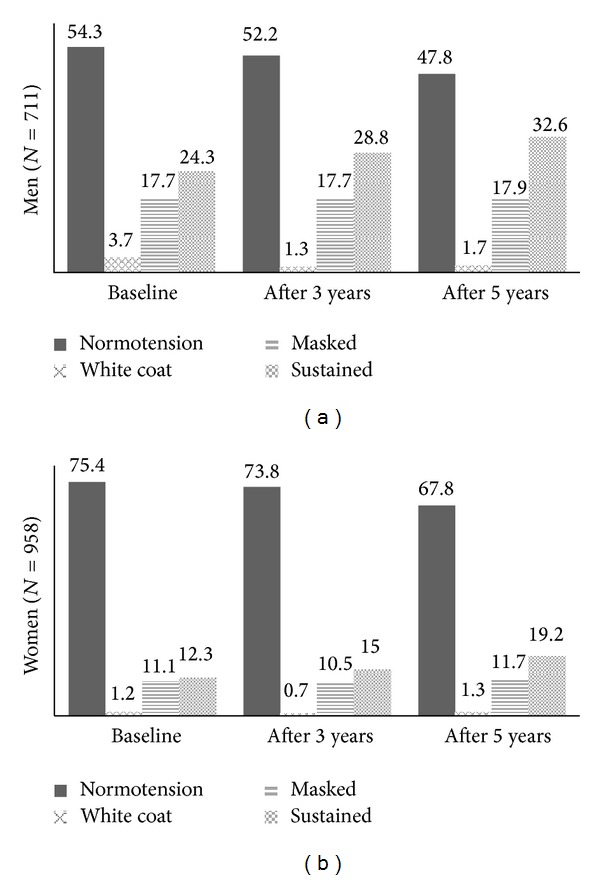
Prevalence of hypertension categories over time for all study participants (*N* = 1669).

Figure 2 presents the prevalence of hypertension categories over time, for all study participants (*N* = 1669). Normotension prevalence has decreased among men (from 54.3% to 47.8%) and women (75.4% to 67.8%). Conversely, sustained hypertension prevalence has increased among both genders (from 24.3% to 32.6% in men and from 12.3% to 19.2% in women). MH prevalence remained stable across all three measurement periods among men (ranging from 17.7% to 17.9%) and women (ranging from 10.5 to 11.7%). The proportion of sustained hypertensives who were classified as such because of medication for hypertension rose from 35.3% to 65.5% and from 53.4% to 74.5% between baseline and last follow-up, in men and women, respectively (not shown).

**Figure 3:**
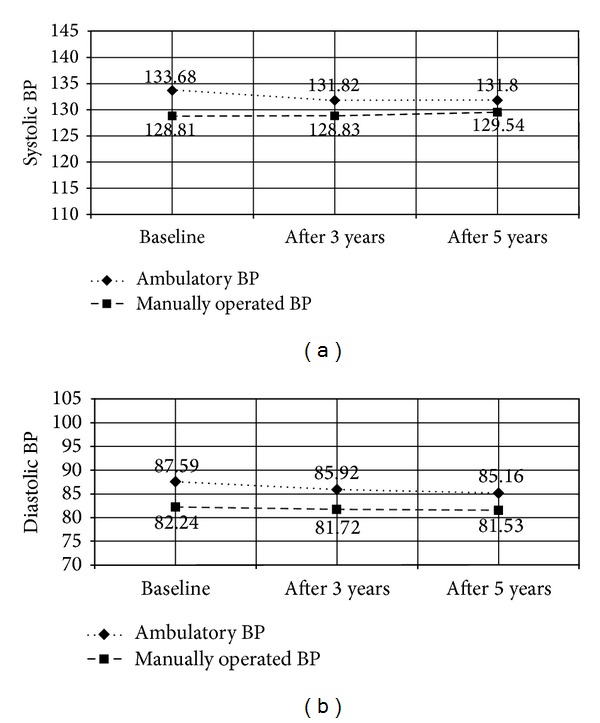
Baseline masked hypertensives ambulatory and manually operated blood pressure means over time (*N* = 232).

Figure 3 presents MOBP and ABP means over time, among baseline masked hypertensives (*N* = 232). ABP means were consistently higher than MOBP means across all three measurement times. However, differences between ABP and MOBP decreased from 4.87 to 2.99 mmHg (systolic) and from 5.35 to 4.2 mmHg (diastolic) after 3 years.

Table 3 presents the evolution of hypertension categories, after 3 years, among those 126 men and 106 women who were masked hypertensives at baseline. MH persistence was 37% and 39%, among men and women, respectively. Among these baseline masked hypertensives, 32 men (25%) and 29 women (27%) had developed sustained hypertension, after 3 years. When considering hypertension, either masked or sustained, 62% of men and 66% of women with MH at baseline had the condition after 3 years.

Table 4 presents the evolution of hypertension categories, after 5 years. Among participants with masked hypertension at baseline, the overall persistence of masked hypertension was 34.1% in men and 33% in women. Progression to sustained hypertension was observed for 31% and 39.6% of these baseline masked hypertensives. Among participants with masked hypertension both at baseline and after 3 years, the persistence of MH, from 3 to 5 years, was present in 43 participants out of 88, who already had the condition for a prolonged period of time (22 men and 21 women). Therefore, the within-subject persistence of masked hypertension after 5 years was 18.5% (43/232). Progression to sustained hypertension, within 5 years, occurred in 37% of baseline masked hypertensives (85/232). Among participants with masked hypertension both at baseline and after 3 years, hypertension, either masked or sustained, was present in 72.3% of men and 80.5% of women after 5 years.

## 4. Discussion

MH prevalence remained stable across all three measurement periods, among all study participants. Persistence of MH, however, decreased over time (from 38% after 3 years to 18.5% after 5 years). In the present study, persistence rather than reproducibility was chosen to describe the evolution of MH, as persistence more closely refers to the natural history of the condition. In this perspective, participant with MH at baseline could be free of this condition after 3 years, not because MH is not reproducible but because changes occurred in their characteristics, environment, or clinical management.

In the literature, MH has been hypothesized to be a transient status which progresses to sustained (and detectable) hypertension when a sufficient time lapse has occurred [16]. The present study provides some evidence supporting this hypothesis, as about 37% of baseline masked hypertensives progressed to sustained hypertension over time. The study however also demonstrates that a significant proportion of baseline masked hypertensives remained as such after 5 years. It argues in favor of MH being an important clinical issue: about one-third of masked hypertensives progressed to sustained hypertension, and about one out of five remained masked after 5 years. Finally, the results of the present study also suggest that persistent masked hypertensives might be more likely to have the condition over a prolonged period of time, as one-half of participants with masked hypertension both at baseline and after 3 years still had MH after 5 years.

Among baseline masked hypertensives, mean ABP values were lower by the years, while MOBP was stable. Two reasons might explain why mean ABP values were lower. Firstly, about 1/3 of these baseline MH regressed to normotension from baseline to 3 years. Secondly, a proportion of those baseline masked hypertensives who progressed to sustained hypertension were treated, lowering their BP values. The stability of MOBP could be attributable to a lower precision caused by the limited number of measurements, compared to ABP.

Our study has a number of strengths including its high participation rate at baseline, its high retention rate over 5 years, and its sizeable sample size, compared to previous studies on this topic [17], for both genders. Also, in our study, both MOBP and ABP were assessed using the same oscillometric device in the same environmental settings (i.e., the workplace). Methods used to identify MH in previous studies generally imply different devices, which could have influenced prevalence. Finally, the present study examined MH persistence in a sizeable cohort of workers over 5 years, using a 3-time measurement design. To our knowledge, this has never been done previously.

Our study also has limitations. First, as stated earlier, participants were systematically informed about their hypertensive status between measurements and referred to their physician when their ABP values were compatible with the diagnosis of hypertension. This screening effect might have led to underestimation of MH persistence to the profit of sustained hypertension given that a proportion of baseline masked hypertensives could have sought medical care for their otherwise unknown condition and therefore be classified as sustained hypertensive at follow-ups. In the present study, MH persistence estimate was nonetheless within the range of previous reports [4, 5]. It should however be noted that comparability of these estimates across studies is not straightforward, given the heterogeneous populations, the variety of procedure used to measure BP, and the different lengths of follow-up. The persistence of MH could also have been underestimated due to a selection bias in loss at follow-up. Although no significant difference was found between losses and participants regarding hypertension classification at baseline, losses at follow-up had higher systolic and diastolic MOBP at baseline (126.4/79.0 mmHg versus 124.6/77.8 mmHg, *P* < 0.05), as well as higher systolic ABP (126.2 versus 125.0 mmHg, *P* < 0.05). They also had a higher body mass index (mean BMI = 27.0 versus 25.0 kg/m^2^). This might suggest that workers who were lost had a worse cardiovascular profile than study participants which could have underestimated the persistence of MH. However, the retention rate was high (77%), limiting the possibility of an important underestimation of MH persistence. Finally, the external validity of this study is potentially limited due to the fact that the population was entirely composed of white-collar workers. Further research is needed to investigate the extent to which our findings are generalized to other employees and other populations.

It is also of interest to note that white coat hypertension prevalence was very low. As mentioned in a previous article [18], the low prevalence of white coat hypertension may partially be attributable to the method used to collect MOBP measurements and characteristics of the population being studied. Indeed, MOBP measurements were made by a trained technician, instead of a physician, and at work, rather than in a medical environment. Also, white coat hypertension is more often detected in older people, and one of the predictors of a positive white coat effect has been previously given a diagnosis of hypertension [19].

In the present study, the prevalence of MH at baseline, 11% in women and 18% in men, was within the range of previous reports [17, 20]. This prevalence, applied to the adult middle-aged population, might amount to more than 20 million people in the case of the United States [1]. Considering that one masked hypertensive out of three might evolve toward sustained hypertension and one out of five may remain masked after 5 years, this could translate in more than 10 million people for whom diagnosis and treatment would be delayed. This estimate is most likely underestimated considering that participants were referred to their physician when their ABP values, at baseline and at 3 years, were compatible with the diagnosis of hypertension. As for persistent masked hypertensives, many questions remain to be answered regarding their cardiovascular risk profile. More studies are needed to clarify this issue and to determine whether improvement in MH screening could lead to beneficial effect on CVD morbidity and mortality.

## 5. Conclusion

Among baseline masked hypertensives, hypertension, either masked or sustained, was present in more than half after 5 years, as one-third progressed to sustained hypertension and about one out of five remained masked. These individuals might be at particular risk for cardiovascular disease because of delays in diagnosis and treatment.

## Figures and Tables

**Table 1:** Description of the study population at baseline.

	Normotension	White coat hypertension	Masked hypertension	Sustained hypertension	*P*
Gender					<0.001
Women	722 (75.4)	12 (1.3)	106 (11.1)	118 (12.3)	
Men	386 (54.3)	26 (3.7)	126 (17.7)	173 (24.3)	
Age					<0.001
<40	321 (75.9)	13 (3.07)	57 (13.5)	32 (7.6)	
40–49	583 (68.2)	17 (2.0)	115 (13.5)	140 (16.4)	
≥50	204 (52.2)	8 (2.1)	60 (15.4)	119 (30.4)	
Education					0.0209
Less than college	287 (66.9)	9 (2.1)	43 (10.0)	90 (21.0)	
College	340 (69.4)	9 (1.8)	72 (14.7)	69 (14.1)	
University	481 (64.1)	20 (2.7)	117 (15.6)	132 (17.6)	
Sedentary behaviours					0.6673
No	950 (66.3)	35 (2.4)	201 (14.0)	247 (17.3)	
Yes	153 (66.2)	3 (1.3)	31 (13.4)	44 (19.1)	
Body mass index (kg/m^2^)					<0.001
<25	589 (76.1)	11 (1.4)	93 (12.0)	81 (10.5)	
25–26.9	196 (64.3)	10 (3.3)	45 (14.8)	54 (17.7)	
≥27	319 (54.6)	17 (2.9)	93 (15.9)	155 (26.5)	
Family history of CVD					0.0062
No	760 (69.0)	25 (2.3)	146 (13.3)	171 (15.5)	
Yes	314 (60.9)	12 (2.3)	77 (14.9)	113 (21.9)	
Alcohol intake					0.0003
<1/week	369 (68.1)	15 (2.8)	63 (11.6)	95 (17.5)	
1–5/week	503 (70.5)	13 (1.8)	89 (12.5)	108 (15.2)	
≥6/week	236 (57.2)	10 (2.4)	79 (19.1)	88 (21.3)	
Smoking status					0.7179
No	978 (66.7)	34 (2.3)	204 (13.9)	250 (17.1)	
Yes	130 (64.3)	4 (2.0)	27 (13.4)	41 (20.3)	

Missing values range from 0 to 3.8%. Sociodemographic variables (gender, age, and education) had no missing value.

**Table 2:** Clinical characteristics at baseline according to hypertension category.

	Normotension	White coat hypertension	Masked hypertension	Sustained hypertension
Men (*N* = 711)	*N* = 386	*N* = 26	*N* = 126	*N* = 173

Manually operated blood pressure (mmHg)				
Systolic	123.0 ± 8	141.8 ± 5	130.1 ± 6	142.9 ± 12
Diastolic	75.5 ± 6	86.2 ± 7	82.5 ± 5	89.5 ± 9
Pulse pressure	47.5 ± 7	55.7 ± 8	47.7 ± 6	53.4 ± 9
Ambulatory blood pressure (mmHg)				
Systolic	123.3 ± 6	129.4 ± 4	134.6 ± 6	139.8 ± 11
Diastolic	77.9 ± 4	80.0 ± 3	87.8 ± 4	89.6 ± 8
Pulse pressure	45.4 ± 5	49.4 ± 4	46.8 ± 6	50.2 ± 7

Women (*N* = 958)	*N* = 722	*N* = 12	*N* = 106	*N* = 118

Manually operated blood pressure (mmHg)				
Systolic	116.7 ± 9	138.9 ± 8	127.4 ± 7	143.4 ± 14
Diastolic	73.0 ± 7	88.7 ± 5	82.1 ± 5	92.8 ± 10
Pulse pressure	43.7 ± 6	50.2 ± 12	45.3 ± 7	50.6 ± 12
Ambulatory blood pressure (mmHg)				
Systolic	118.1 ± 7	126.5 ± 6	132.8 ± 7	140.6 ± 13
Diastolic	75.7 ± 5	80.1 ± 3	87.4 ± 4	91.5 ± 10
Pulse pressure	42.3 ± 5	46.4 ± 6	45.3 ± 7	49.1 ± 9

One hundred and twenty-three (53%) of masked hypertensives were diagnosed from diastolic BP only. Twenty eight (12%) of masked hypertensives were diagnosed from systolic BP only. Eighty-one (35%) of masked hypertensives were diagnosed from both BP types.

**Table 3:** Hypertension categories evolution from baseline to 3 years among participants with masked hypertension at baseline (*N* = 232).

	*N*	Regression to normotension	Transition to white coat hypertension	Persistence of masked hypertension	Progression to sustained hypertension
Men	126	47 (37.3%)	0	47 (37.3%)	32 (25.4%)
Women	106	35 (33%)	1 (0.9%)	41 (38.7%)	29 (27.4%)

Total	232	82 (35.3%)	1 (0.5%)	88 (37.9%)	61 (26.3%)

**Table 4:** Hypertension categories evolution after 5 years.

	*N*	Regression to normotension	Transition to white coat hypertension	Persistence of masked hypertension	Progression to sustained hypertension
From baseline to 5 years (*N* = 232)					
Men	126	43 (34.1%)	1 (0.8%)	43 (34.1%)	39 (31%)
Women	106	29 (27.4%)	0	35 (33%)	42 (39.6%)

Total	232	72 (31.0%)	1 (0.5%)	78 (33.6%)	81 (34.9%)

Within-subject masked hypertension persistence (*N* = 88)*					
Men	47	13 (27.7%)	0	22 (46.8%)	12 (25.5%)
Women	41	8 (19.5%)	0	21 (51.2%)	12 (29.3%)

Total	88	21 (23.9%)	0	43 (48.8%)	24 (27.3%)

*Hypertension categories evolution from 3 to 5 years among participants with masked hypertension both at baseline and after 3 years.
